# Supplementary material for: Experimental exposure to urban and pink noise affects brain development and song learning in zebra finches (Taenopygia guttata)
Source: PeerJ. 2016 Aug 16;4:e2287. doi: 10.7717/peerj.2287 (PMC4991897; doi:10.7717/peerj.2287)
Supplement: Table S1 — Bayesian model estimates for the effect of treatment on baseline circulating corticosterone in juvenile zebra finches. Table S2. Bayesian model estimates for the effect of treatment on post-hatch day 40 song characteristics in juvenile zebra finches. Table S3. Bayesian model estimates for the effect of treatment on the similarity of post-hatch day 60 song characteristics to crystallized song characteristics (PHD 100) in juvenile zebra finches. Table S4. Bayesian model estimates for the effect of treatment on crystallized song characteristics (PHD 100 - Group 1) of juvenile zebra finches. Table S5. Bayesian model estimates for the effect of treatment (noise versus silent only) on crystallized song characteristics (PHD 200 - Group 2) of juvenile zebra finches. Table S6. Bayesian model estimates for the effect of treatment on brain structure volumes (relative to total brain size) of juvenile ( PHD100; group 1) zebra finches. Table S7a. DIC comparison table for Bayesian models predicting song similarity to tutor. Table S7b. Bayesian model estimates for the models with lowest DIC score in the comparison for predicting similarity to tutor song. Table S8. Bayesian model estimates for the effect of treatment on song similarity to tutor of juvenile ( PHD200; group 2) zebra finches. [file peerj-04-2287-s002.docx]

Table S1. Bayesian model estimates for the effect of treatment on post-hatch day 40 song characteristics in juvenile zebra finches

| **Node** | **Mean** | **SD** | **2.50%** | **Median** | **97.50%** |
| --- | --- | --- | --- | --- | --- |
|  |  |  |  |  |  |
| **Minimum Frequency** | 395 | 53.58 | 292.2 | 393.9 | 503.8 |
| Treatment effect | 37.51 | 25.19 | -14.4 | 38.43 | 84.72 |
| Nest effect (standard deviation) | 63.53 | 23.09 | 9.821 | 66.46 | 97.81 |
|  |  |  |  |  |  |
| **Maximum Frequency** | 11970 | 67.74 | 11840 | 11970 | 12100 |
| Treatment effect | -258 | 31.62 | -320.3 | -258 | -196.1 |
| Nest effect (standard deviation) | 99.86 | 0.1413 | 99.48 | 99.9 | 100 |
|  |  |  |  |  |  |
| **Percentage of fully developed syllables** | -0.4073 | 2.79 | -5.952 | -0.4062 | 5.136 |
| Treatment effect | 1.379 | 1.253 | -1.148 | 1.397 | 3.821 |
| Nest effect (standard deviation) | 2.062 | 1.459 | 0.1035 | 1.822 | 5.514 |

Table S2. Bayesian model estimates for the effect of treatment on the similarity of post-hatch day 60 song characteristics to crystallized song characteristics (PHD 100) in juvenile zebra finches.

| **Node** | **Mean** | **SD** | **2.50%** | **Median** | **97.50%** |
| --- | --- | --- | --- | --- | --- |
|  |  |  |  |  |  |
| **% Similarity** | 55.03 | 10.45 | 34.27 | 55.09 | 75.65 |
| Treatment effect | 2.978 | 4.881 | -6.582 | 2.913 | 12.85 |
| Nest effect (standard deviation) | 16.94 | 5.533 | 7.355 | 16.39 | 29.52 |
|  |  |  |  |  |  |
| **% Accuracy** | 69.33 | 1.551 | 66.31 | 69.31 | 72.46 |
| Treatment effect | 0.9008 | 0.6675 | -0.4588 | 0.9125 | 2.19 |
| Nest effect (standard deviation) | 1.144 | 0.8806 | 0.05243 | 0.9623 | 3.275 |
|  |  |  |  |  |  |
| **% Sequential similarity** | 54.21 | 7.763 | 38.56 | 54.28 | 69.52 |
| Treatment effect | 3.471 | 3.549 | -3.453 | 3.393 | 10.79 |
| Nest effect (standard deviation) | 10.05 | 5.318 | 0.9707 | 9.852 | 21.41 |
|  |  |  |  |  |  |
| **Pitch difference** | 1.587 | 0.3471 | 0.8881 | 1.589 | 2.276 |
| Treatment effect | -0.054 | 0.1633 | -0.3786 | -0.05561 | 0.2745 |
| Nest effect (standard deviation) | 0.5898 | 0.1838 | 0.2656 | 0.5716 | 1.005 |

Table S3. Bayesian model estimates for the effect of treatment on crystallized song characteristics (PHD 100 - Group 1) of juvenile zebra finches.

| **Node** | **Mean** | **SD** | **2.50%** | **Median** | **97.50%** |
| --- | --- | --- | --- | --- | --- |
|  |  |  |  |  |  |
| **Minimum frequency** | 595.2 | 53.88 | 490.1 | 594.8 | 702.3 |
| Treatment effect | 1.59 | 24.38 | -46.72 | 1.778 | 49.29 |
| Nest effect (standard deviation) | 78.11 | 15.75 | 41.1 | 80.82 | 99.02 |
|  |  |  |  |  |  |
| **Maximum frequency** | 18710 | 67.15 | 18580 | 18710 | 18840 |
| Treatment effect | -874.9 | 30.59 | -934.8 | -874.9 | -814.7 |
| Nest effect (standard deviation) | 99.94 | 0.05958 | 99.78 | 99.96 | 100 |
|  |  |  |  |  |  |
| **Peak frequency** | 4475.0 | 67.06 | 4344.0 | 4475.0 | 4607.0 |
| Treatment effect | -69.81 | 30.49 | -129.5 | -69.79 | -9.972 |
| Nest effect (standard deviation) | 99.65 | 0.3514 | 98.7 | 99.76 | 99.99 |
|  |  |  |  |  |  |
| **Complexity (# notes)** | 5.593 | 1.028 | 3.491 | 5.613 | 7.575 |
| Treatment effect | -0.3364 | 0.4455 | -1.218 | -0.3383 | 0.5603 |
| Nest effect (standard deviation) | 0.971 | 0.6597 | 0.03884 | 0.8906 | 2.48 |
|  |  |  |  |  |  |
| **Duration** | 0.8112 | 0.1601 | 0.4883 | 0.8126 | 1.126 |
| Treatment effect | -0.006857 | 0.07028 | -0.1483 | -0.00641 | 0.1319 |
| Nest effect (standard deviation) | 0.153 | 0.1021 | 0.00839 | 0.1398 | 0.3858 |
|  |  |  |  |  |  |
| **Tempo** | 6.851 | 0.7638 | 5.291 | 6.865 | 8.328 |
| Treatment effect | -0.2918 | 0.3356 | -0.9427 | -0.2979 | 0.3939 |
| Nest effect (standard deviation) | 0.7977 | 0.4811 | 0.0617 | 0.7558 | 1.87 |

Table S4. Bayesian model estimates for the effect of treatment (noise versus silent only) on crystallized song characteristics (PHD 200 - Group 2) of juvenile zebra finches.

| **Node** | **Mean** | **SD** | **2.50%** | **Median** | **97.50%** |
| --- | --- | --- | --- | --- | --- |
| **Minimum frequency** | 659.6 | 89.96 | 479.6 | 660 | 837.7 |
| Treatment effect | 17.79 | 53.78 | -88.58 | 17.75 | 125.6 |
| Nest effect (standard deviation) | 52.82 | 26.36 | 3.734 | 55.56 | 96.26 |
|  |  |  |  |  |  |
| **Maximum frequency** | 10520 | 123.2 | 10270 | 10530 | 10760 |
| Treatment effect | 120.3 | 73.33 | -17.65 | 117.7 | 271.1 |
| Nest effect (standard deviation) | 74.7 | 22.84 | 13.13 | 81.73 | 99.31 |
|  |  |  |  |  |  |
| **Peak frequency** | 4410 | 149.2 | 4117 | 4409 | 4703 |
| Treatment effect | -229.1 | 89.38 | -404.3 | -228.9 | -54.08 |
| Nest effect (standard deviation) | 99.34 | 0.6591 | 97.57 | 99.54 | 99.98 |
|  |  |  |  |  |  |
| **Complexity** | 4.284 | 1.458 | 1.395 | 4.272 | 7.204 |
| Treatment effect | 1.434 | 0.9224 | -0.402 | 1.435 | 3.261 |
| Nest effect (standard deviation) | 0.885 | 0.5592 | 0.05157 | 0.8527 | 2.134 |
|  |  |  |  |  |  |
| **Duration** | 0.8456 | 0.2577 | 0.325 | 0.8455 | 1.361 |
| Treatement effect | 0.03685 | 0.1635 | -0.2874 | 0.03664 | 0.366 |
| Nest effect (standard deviation) | 0.1526 | 0.0973 | 0.008123 | 0.1471 | 0.369 |
|  |  |  |  |  |  |
| **Tempo** | 5.476 | 1.083 | 3.31 | 5.473 | 7.639 |
| Treatment effect | 0.1232 | 0.6847 | -1.25 | 0.1248 | 1.496 |
| Nest effect (standard deviation) | 0.6511 | 0.4046 | 0.03897 | 0.6261 | 1.561 |

Table S5. Bayesian model estimates for the effect of treatment on baseline circulating corticosterone in juvenile zebra finches

| **Node** | **Mean** | **SD** | **2.50%** | **Median** | **97.50%** |
| --- | --- | --- | --- | --- | --- |
| Cort | 3.19 | 0.5731 | 2.056 | 3.191 | 4.334 |
| Treatment effect | 0.0612 | 0.2512 | -0.4485 | 0.06455 | 0.5512 |
| Nest effect (standard deviation) | 0.5276 | 0.3432 | 0.02867 | 0.4892 | 1.306 |

Table S6. Bayesian model estimates for the effect of treatment on brain structure volumes (relative to total brain size) of juvenile (~PHD100; group 1) zebra finches.

| **Node** | **Mean** | **SD** | **2.50%** | **Median** | **97.50%** |
| --- | --- | --- | --- | --- | --- |
| **RA volume** | 0.215 | 0.007445 | 0.2003 | 0.215 | 0.2297 |
| Treatment effect | -0.001047 | 0.007268 | -0.01531 | -0.001053 | 0.01336 |
| Total brain volume effect | -2.43E-04 | 5.05E-04 | -0.001242 | -2.40E-04 | 7.55E-04 |
| No. brothers effect | -5.32E-04 | 0.008174 | -0.01678 | -4.88E-04 | 0.01559 |
|  |  |  |  |  |  |
| **HVC volume** | 0.2853 | 0.01209 | 0.2615 | 0.2853 | 0.3092 |
| Treatment effect | -0.03381 | 0.01182 | -0.05713 | -0.03383 | -0.01039 |
| Total brain volume effect | -6.09E-04 | 8.20E-04 | -0.002227 | -6.07E-04 | 0.001012 |
| No. brothers effect | 0.03551 | 0.01327 | 0.009179 | 0.03555 | 0.06175 |
|  |  |  |  |  |  |
| **Area X volume** | 1.09 | 0.0621 | 0.9678 | 1.09 | 1.213 |
| Treatment effect | -0.1101 | 0.06448 | -0.2372 | -0.1101 | 0.01743 |
| Total brain volume effect | -0.002055 | 0.004411 | -0.01077 | -0.002035 | 0.006652 |
| No. brothers effect | 0.1742 | 0.07402 | 0.02698 | 0.1746 | 0.3204 |

Table S7a. DIC comparison table for Bayesian models predicting song similarity to tutor.

|  | MODEL | DIC |
| --- | --- | --- |
| **% Similarit**y |  |  |
|  | Treatment & Brothers | 220.643 |
|  | Treatment & Total brain size & Brothers | 224.199 |
|  | Treatment & HVC & Brothers | 222.903 |
|  | Treatment & RA & Brothers | 223.15 |
|  | Treatment & Area X & Brothers | 217.225 |
|  | Treatment & HVC & Area X & Brothers | 223.017 |
|  | Treatment & RA & Area X & Brothers | 223.017 |
|  | Treatment & HVC & RA & Brothers | 223.835 |
|  | Treatment & HVC & RA & Area X & Brothers | 219.782 |
|  |  |  |
| **Accuracy** |  |  |
|  | Treatment & Brothers | 198.639 |
|  | Treatment & Total brain size & Brothers | 203.25 |
|  | Treatment & HVC & Brothers | 203.006 |
|  | Treatment & RA & Brothers | 203.152 |
|  | Treatment & Area X & Brothers | 196.356 |
|  | Treatment & HVC & Area X & Brothers | 204.951 |
|  | Treatment & RA & Area X & Brothers | 204.951 |
|  | Treatment & HVC & RA & Brothers | 203.843 |
|  | Treatment & HVC & RA & Area X & Brothers | 198.172 |
|  |  |  |
| **% Sequential** |  |  |
|  | Treatment & Brothers | 223.039 |
|  | Treatment & Total brain size & Brothers | 229.626 |
|  | Treatment & HVC & Brothers | 229.286 |
|  | Treatment & RA & Brothers | 229.217 |
|  | Treatment & Area X & Brothers | 219.638 |
|  | Treatment & HVC & Area X & Brothers | 228.275 |
|  | Treatment & RA & Area X & Brothers | 228.275 |
|  | Treatment & HVC & RA & Brothers | 229.961 |
|  | Treatment & HVC & RA & Area X & Brothers | 222.311 |

Table S7b. Bayesian model estimates for the models with lowest DIC score in the comparison for predicting similarity to tutor song.

| **Node** | **Mean** | **SD** | **2.50%** | **Median** | **97.50%** |
| --- | --- | --- | --- | --- | --- |
| **Sequential similarity** | 83.11 | 7.658 | 68.01 | 83.09 | 98.3 |
| Treatment effect | -7.366 | 5.765 | -18.75 | -7.375 | 4.13 |
| No. brothers effect | 3.507 | 6.642 | -9.637 | 3.518 | 16.61 |
| Treatment*brother interaction | -6.87 | 4.828 | -16.49 | -6.876 | 2.666 |
| Area X Volume effect | -28.24 | 23.45 | -74.86 | -28.35 | 18.19 |
| Area X Volume*brother interaction | 19.07 | 33.08 | -46.98 | 19.21 | 84.45 |
| Area X* Treatment interaction | -11.92 | 35.89 | -82.94 | -11.98 | 59.47 |
|  |  |  |  |  |  |
| **Accuracy** | 73.85 | 4.618 | 64.74 | 73.84 | 83.01 |
| Treatment effect | -1.791 | 3.475 | -8.651 | -1.797 | 5.139 |
| No. brothers effect | -2.779 | 4.004 | -10.7 | -2.773 | 5.123 |
| Treatment*brother interaction | -6.664 | 2.911 | -12.46 | -6.668 | -0.9155 |
| Area X Volume effect | 4.01 | 14.14 | -24.1 | 3.944 | 32 |
| Area X Volume*brother interaction | -0.2575 | 19.96 | -40.11 | -0.177 | 39.18 |
| Area X* Treatment interaction | 1.557 | 21.65 | -41.3 | 1.52 | 44.62 |
|  |  |  |  |  |  |
| **Similarity** | 61.9 | 7.267 | 47.56 | 61.88 | 76.31 |
| Treatment effect | -2.103 | 5.47 | -12.9 | -2.112 | 8.804 |
| No. brothers effect | -6.241 | 6.303 | -18.71 | -6.231 | 6.197 |
| Treatment*brother interaction | -6.169 | 4.582 | -15.3 | -6.175 | 2.879 |
| Area X Volume effect | 3.29 | 22.25 | -40.95 | 3.185 | 47.34 |
| Area X Volume*brother interaction | -18.85 | 31.39 | -81.52 | -18.73 | 43.2 |
| Area X* Treatment interaction | 11.3 | 34.06 | -56.12 | 11.25 | 79.03 |

Table S8. Bayesian model estimates for the effect of treatment on song similarity to tutor of juvenile (~PHD200; group 2) zebra finches.

| **Node** | **Mean** | **SD** | **2.50%** | **Median** | **97.50%** |
| --- | --- | --- | --- | --- | --- |
| % Similarity | 67.34 | 24.84 | 18.18 | 67.11 | 117.1 |
| Treatment effect | 0.7418 | 15.71 | -30.81 | 0.8696 | 31.77 |
| Nest effect (standard deviation) | 14.78 | 9.397 | 0.7103 | 14.27 | 35.8 |
|  |  |  |  |  |  |
| Accuracy | 73.81 | 5.213 | 63.49 | 73.84 | 84.13 |
| Treatment effect | 3.453 | 3.306 | -3.055 | 3.426 | 10.03 |
| Nest effect (standard deviation) | 3.049 | 1.976 | 0.1675 | 2.93 | 7.345 |
|  |  |  |  |  |  |
| % Sequential similarity | 53.45 | 10.26 | 32.84 | 53.43 | 73.79 |
| Treatment effect | 12.05 | 6.497 | -1.055 | 12.09 | 25.07 |
| Nest effect (standard deviation) | 6.166 | 3.901 | 0.3797 | 5.911 | 14.96 |
